# Supplementary material for: Microglia regulate nucleus accumbens synaptic development and circuit function underlying threat avoidance behaviors
Source: Res Sq. 2025 Jan 22:rs.3.rs-5837701. Preprint. [Version 1] doi: 10.21203/rs.3.rs-5837701/v1 (PMC11838711; doi:10.21203/rs.3.rs-5837701/v1)
Supplement: Supplement 1 [file NIHPPrs5837701v1-supplement-1.pdf]

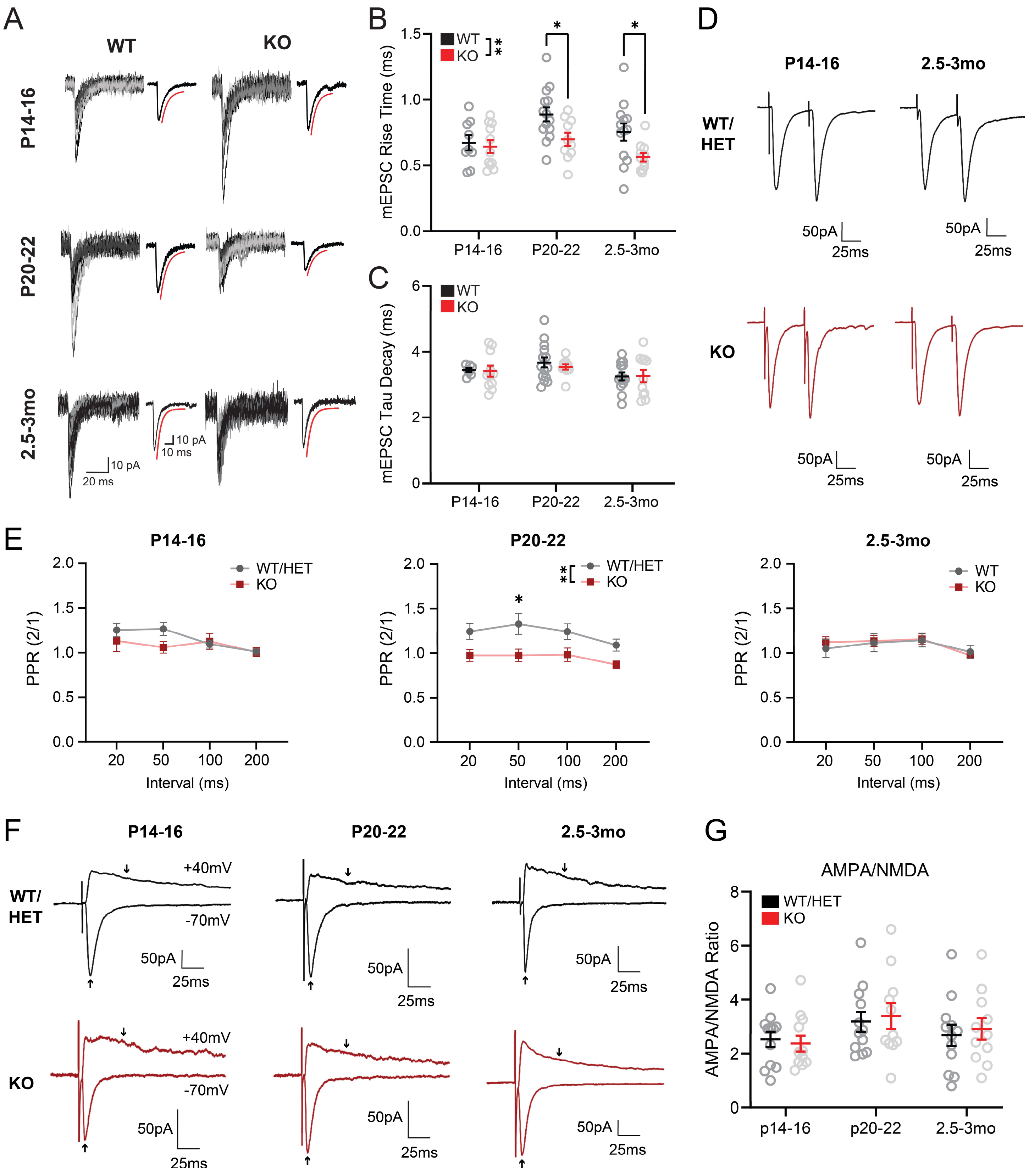

Figure S1: Additional electrophysiological measures of excitatory synaptic development in the presence or absence of microglia. (A) Overlay of 10 to 15 single mEPSC events in WT and KO mice at p14-16, p20-22 and 2.5-3mo. The average trace for each condition is represented on the right, and the fit exponential curve is shown in red. (B) mEPSC rise time in NAc of WT and KO mice at ages with a sufficient number of events for analysis of kinetics. $F_{age}(2,62)=4.268$, $P=0.0183$; $F_{genotype}(1,62)=9.472$, $P=0.0031$; $F_{age\ x\ genotype}(2,62)=1.390$, $P=0.2568$. (C) mEPSC Tau decay constant in NAc of WT and

KO mice. $F_{age}(2,60)=3.009$, $P=0.0569$; $F_{genotype}(1,60)=0.1783$, $P=0.6743$; $F_{age \times genotype}(2,60)=0.1469$, $P=0.8637$. Two-way ANOVA with Šídák's multiple comparisons test. P14-16: WT *n*=9(6), KO *n*=11(5); P20-22: WT *n*=14(7), KO *n*=10(6); 2.5-3mo: WT *n*=13(7), KO *n*=11(6). (D) Representative traces of evoked paired-pulse ratio (PPR) recordings with a 50ms interstimulus interval in NAc knockout and control mice at P14-16 and 2.5-3mo. Cells were recorded while voltage clamping at -70mV in the presence of 100µM picrotoxin. (E) Paired-pulse ratio from interstimulus intervals ranging from 20 to 200ms. P14-16: $F_{Interval}(3,69)=5.018$, $P=0.0033$; $F_{Genotype}(1,23)=0.8535$, $P=0.3652$; $F_{Interval \times Genotype}(3,69)=2.294$, $P=0.0855$. P20-22: $F_{Interval}(3,60)=5.807$, $P=0.0240$; $F_{Genotype}(1,20)=5.962$, $P=0.0015$; $F_{Interval \times Genotype}(3,60)=0.8502$, $P=0.4720$. 2.5-3mo: $F_{Interval}(3,69)=8.863$, $P<0.0001$; $F_{Genotype}(1,23)=0.02861$, $P=0.8672$; $F_{Interval \times Genotype}(3,69)=1.001$, $P=0.3976$. Two-way RM ANOVA with Šídák's multiple comparisons test. P14-16: WT/HET *n*=13(4), KO *n*=12(3); P20-22: WT/HET *n*=13(4), KO *n*=12(4); 2.5-3mo: WT *n*=12(4), KO *n*=13(4). (F) Representative traces of evoked AMPA/NMDA recordings in knockout and control mice. Cells were recorded while voltage clamping at -70mV or +40mV in the presence of 100µM picrotoxin. Arrows indicate timepoints used for current estimation: the NMDA current was taken as the current value from the +40mV stimulation 50 ms after stimulus onset, and the AMPA current was taken as the peak amplitude of the evoked current when held at -70mV. (G) AMPA/NMDA ratio in NAc of KO and control mice at P14-16, P20-22, and 2.5-3mo. $F_{Age}(2,64)=2.562$, $P=0.0850$; $F_{Genotype}(1,64)=0.1112$, $P=0.7399$; $F_{Age \times Genotype}(2,64)=0.1802$, $P=0.8355$. Two-way ANOVA. P14-16: WT/HET *n*=12(4), KO *n*=12(3); P20-22: WT/HET *n*=12(5), KO *n*=11(4); 2.5-3mo: WT *n*=12(4), KO *n*=11(4). Error bars represent mean ± SEM. **P*<0.05. ***P*<0.01.

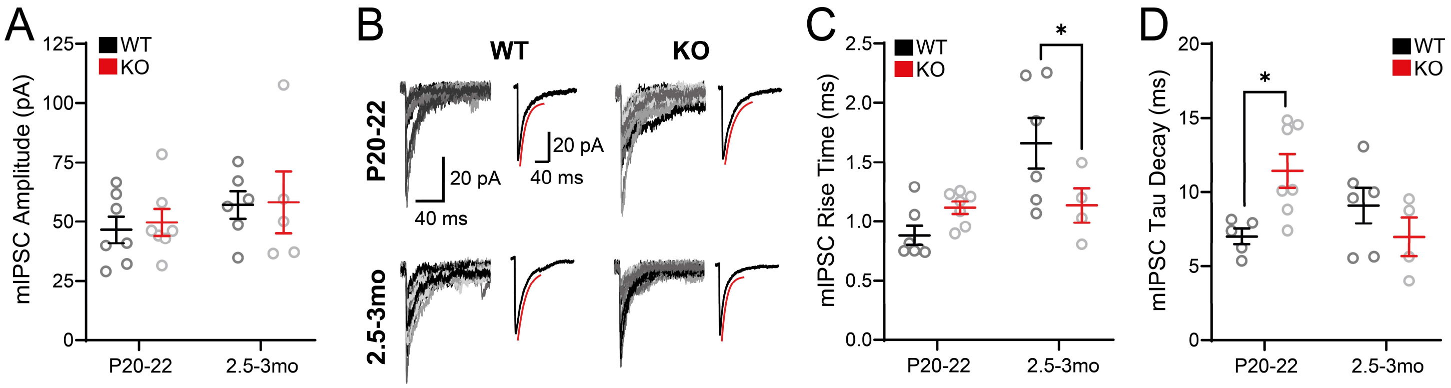

Figure S2: Additional electrophysiological measures of inhibitory synaptic development in the presence or absence of microglia. (A) mIPSC amplitude in NAc of WT and KO mice at P20-22 and 2.5-3mo. $F_{age}(1,21)=1.636$, $P=0.2148$; $F_{genotype}(1,21)=0.08115$, $P=0.7785$; $F_{age\ x\ genotype}(1,21)=0.0169$, $P=0.8978$. (B) Overlay of 10 to 15 single mIPSC events in both WT and KO mice at P20-22 and 2.5-3mo. The average trace for each condition is represented on the right, and the fit exponential curve is shown in red. (C-D) mIPSC rise time (C) and Tau decay constant (D) in NAc of WT and KO mice at P20-22 and 2.5-3mo. Rise time: $F_{age}(1,20)=8.977$, $P=0.0071$; $F_{genotype}(1,20)=1.225$, $P=0.2815$; $F_{age\ x\ genotype}(1,20)=8.159$, $P=0.0098$; Tau Decay: $F_{age}(1,18)=1.074$, $P=0.3137$; $F_{genotype}(1,18)=1.031$, $P=0.3233$; $F_{age\ x\ genotype}(1,18)=8.058$, $P=0.0109$. Two-way ANOVA with Šídák's multiple comparisons test. P20-22: WT n=7(3), KO n=7(3); 2.5-3mo: WT n=6(3), KO n=5(3). *$P<0.05$. Error bars represent mean ± SEM.

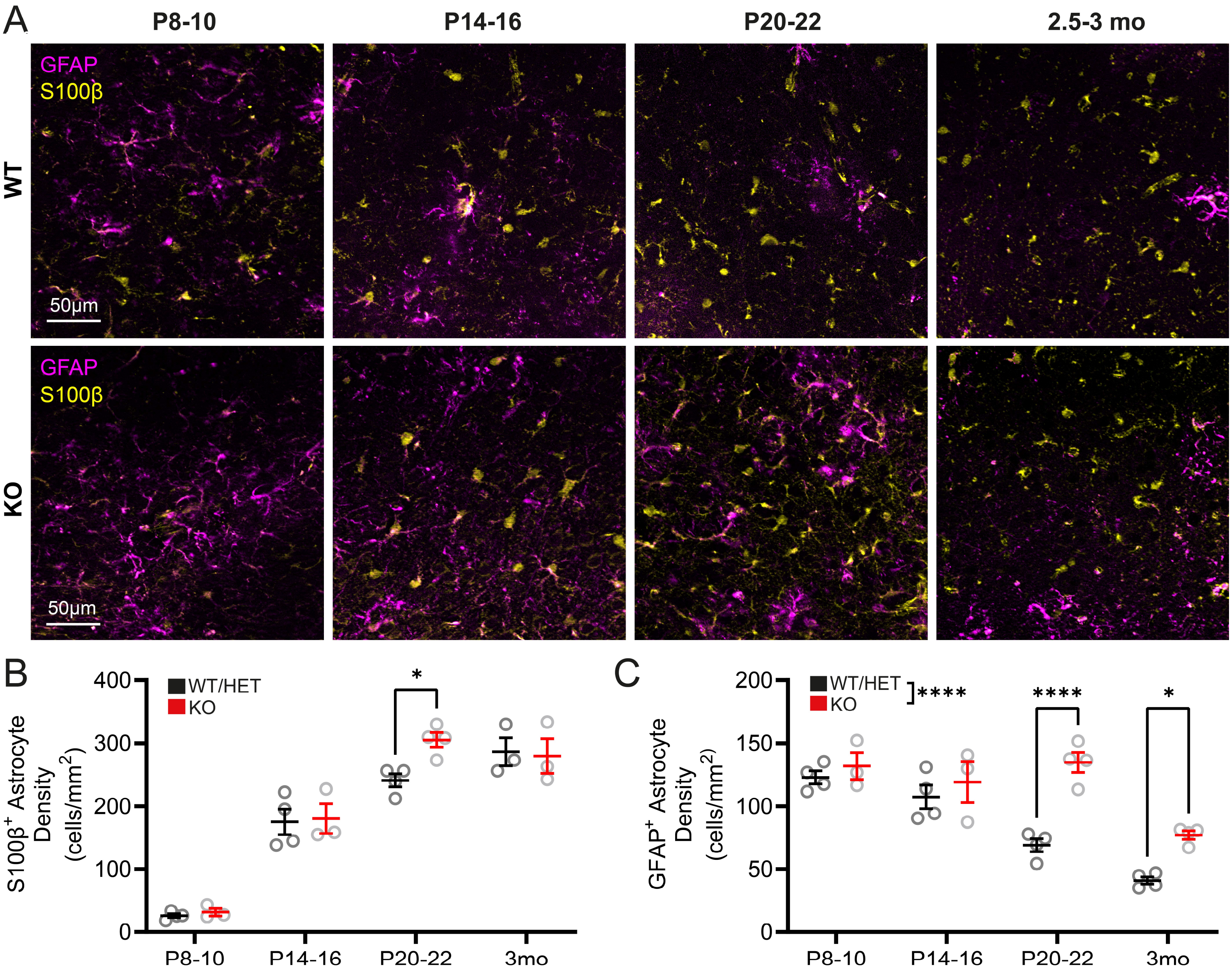

Figure S3: Absence of microglia alters normal developmental trajectories of astrocyte markers in NAc. (A) Representative images of S100β⁺ and GFAP⁺ astrocytes in NAc of WT and KO mice at p8-10, p14-16, p20-22 and 2.5-3mo. Zoom panels represent the area with a dashed outline. (B) S100β⁺ astrocyte density in NAc of WT and KO mice across postnatal development. $F_{age}(3,20)=99.74$, $P<0.0001$; $F_{genotype}(1,20)=2.092$, $P=0.1635$; $F_{age\ x\ genotype}(3,20)=2.004$, $P=0.1458$, Two-way ANOVA with Šídák's multiple comparisons test. P8-10: WT *n*=3, KO *n*=3; P14-16: WT *n*=4, KO *n*=3; P20-22: WT *n*=3, KO *n*=4; 2.5-3mo: WT *n*=3, KO *n*=3. (C) GFAP⁺ astrocyte density in KO and control mice across postnatal development. $F_{age}(3,22)=29.29$, $P<0.0001$; $F_{genotype}(1,22)=30.90$, $P<0.0001$; $F_{age\ x\ genotype}(3,22)=5.798$, $P=0.0045$; Two-way ANOVA with Šídák's multiple comparisons test. P8-10: WT *n*=4, KO *n*=3; P14-16: WT *n*=4, KO *n*=3; P20-22: WT *n*=3, KO *n*=4; 2.5-3mo: WT/HET *n*=4, KO *n*=4. *$P<0.05$, ****$P<0.0001$. Error bars represent mean ± SEM.

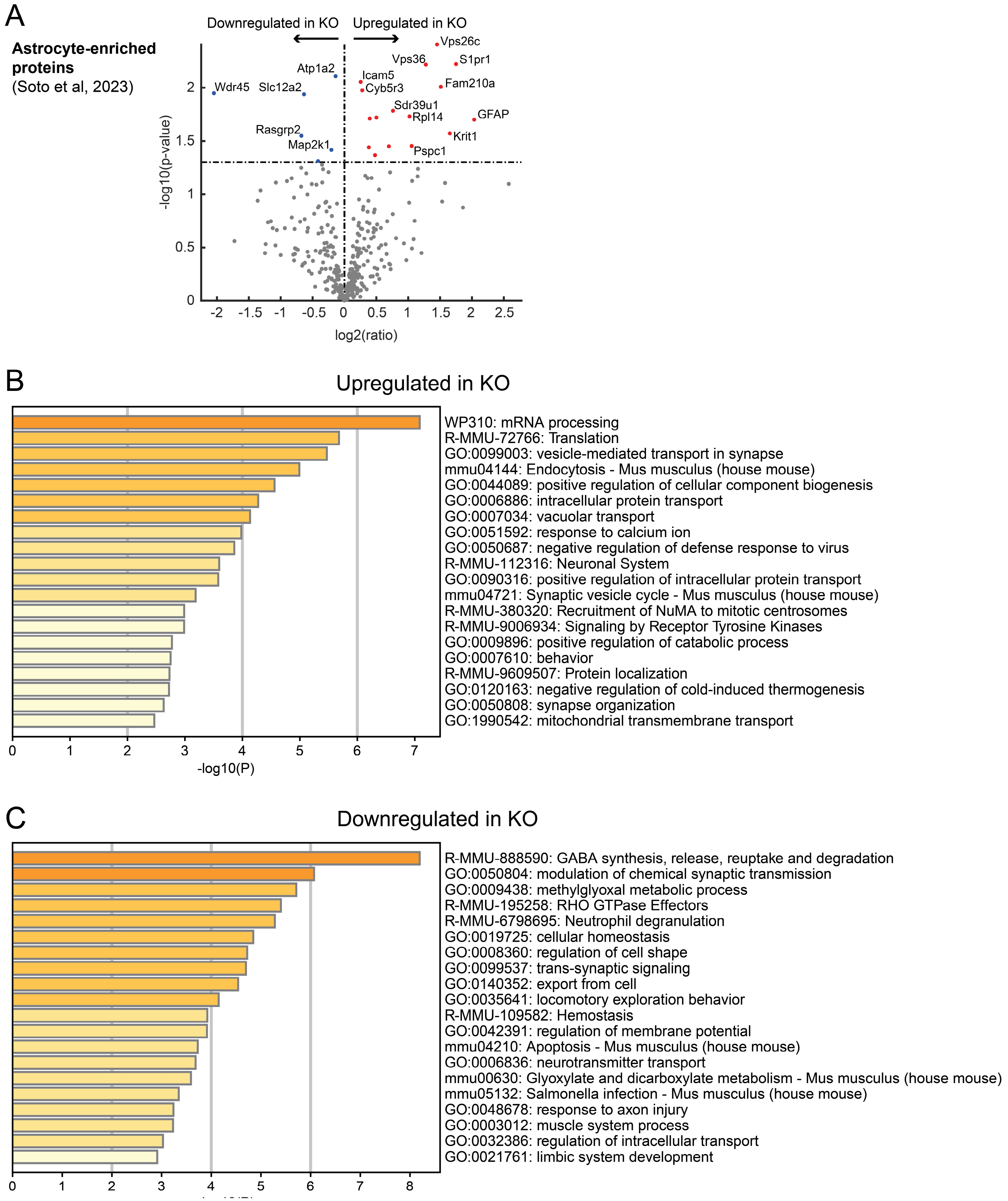

Figure S4. Additional analysis of proteomic dataset. (A) Volcano plot of protein expression in WT vs KO mice in 342 proteins shown to be enriched/unique for astrocytes compared to neurons in Soto et. al. 2023. (B-C) Complete list of top 20 pathways upregulated (B) and downregulated (C) in KO mice upon Metascape analysis of differentially expressed proteins from proteomic data.

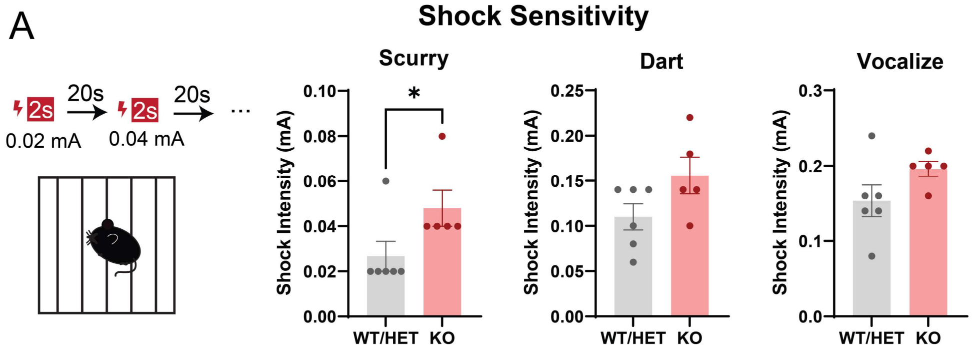

Figure S5. (A) Minimum shock intensity required to elicit scurry, dart, and vocalization responses in knockout and control mice. Mann-Whitney test, WT/HET: *n*=6, KO: *n*=5. *P<0.05. Error bars represent mean ± SEM.
